# A Novel Antibacterial Compound from *Siegesbeckia glabrescens*

**DOI:** 10.3390/molecules171112469

**Published:** 2012-10-24

**Authors:** Young-Soo Kim, Hyungil Kim, Eunsun Jung, Jang-Hyun Kim, Wangtaek Hwang, Eun-Ju Kang, Sanghyun Lee, Byung-Jo Ha, Jongsung Lee, Deokhoon Park

**Affiliations:** 1 Biospectrum Life Science Institute, Eines Platz 11th FL, #442-13 Sangdaewon Dong, Seongnam City, Gyunggi Do 462-120, Korea; 2 Dermiskin, 44-9 Chongho Ri, Jinwi Myeon, Pyeongtaek City, Gyunggi Do 451-862, Korea; 3 Department of Integrative Plant Science, Chung-Ang University, Anseong City, Gyunggi Do 456-756, Korea; 4 Department of Dermatological Health Management, EulJi University, Seongnam City, Gyunggi Do 461-713, Korea

**Keywords:** antibacterial activity, 3-(dodecanoyloxy)-2-(isobutyryloxy)-4-methylpentanoic acid, lauric acid, *Siegesbeckia glabrescens*

## Abstract

The crude methanol extract of the dried aerial parts of *Siegesbeckia glabrescens* (Compositae) showed antibacterial activity against the foodborne pathogen *Staphylococcus aureus*. Bioactivity-guided separation led to the isolation of 3-(dodecanoyloxy)-2-(isobutyryloxy)-4-methylpentanoic acid from nature for the first time.The structure was determined by spectroscopic data analysis (UV, MS, and NMR). The minimal inhibitory concentration (MIC) of 3-(dodecanoyloxy)-2-(isobutyryloxy)-4-methylpentanoic acid against *S. aureus* was found to be 3.12 μg/mL. In addition, in a further antimicrobial activity assay against Gram-positive (*B. subtilis*, *E. faecalis*, *P. acnes*, *S. epidermidis*, *S. schleiferi* subsp. *coagulans*, *S. agalactiae* and *S. pyrogens*), and Gram-negative bacteria (*E. coli* and *P. aeruginosa*), and yeast strains (*C. alibicans* and *F. neoformans*), the antimicrobial activity of the compound was found to be specific for Gram-positive bacteria. The MIC values of the compound for Gram-positive bacteria ranged from 3.12 to 25 μg/mL. Furthermore, it was found that the 2-(isobutyryloxy)-4-methylpentanoic acid substituent may operate as a key factor in the antibacterial activity of the compound, together with the laurate group.

## 1. Introduction

Antimicrobial resistance among Gram-positive bacteria has become increasingly prevalent and has resulted in serious infections worldwide over the past two decades [1,2]. Most nosocomial and community-acquired infections are caused by *Staphylococcus aureus* [3,4,5,6]. Herbs and spices with antibacterial activity have been widely used both traditionally and commercially to increase the shelf-life and safety of foods [7]. With the recent increase in consumer mistrust of synthetic additives, there has been a concomitant increase in the search for new natural compounds from plants that can be used to replace existing synthetic antimicrobials [8].

*Siegesbeckia glabrescens*, well-known as “Hi-Chum” in Korea, is an annual herb that grows in Korea. The aerial portion and roots of *S. glabrescens* have been used as a traditional medicine to treat rheumatic arthritis, asthma, paralysis and allergic disorders. Modern pharmacological experiments showed that the extracts of *S. glabrescens* exhibit antioxidative, antiallergic, antihypertension, antitumor, and anti-inflammatory activities [9]. However, the antibacterial activity of these extracts has not yet been evaluated. Therefore, we investigated the antibacterial effects of the *S. glabrescens* extract and characterized new bioactive compounds from *S. glabrescens*. Herein, we report the isolation, structural identification and antibacterial activity of a new compound, 3-(dodecanoyloxy)-2-(isobutyryloxy)-4-methylpentanoic acid, from *S. glabrescens*.

## 2. Results and Discussion

### 2.1. Isolation of Active Compound from S. glabrescens

The aerial parts of *S. glabrescens* was extracted with 80% MeOH, and fractionated successively with *n*-hexane, CHCl_3_, and EtOAc. The EtOAc extract exhibited high antibacterial activity against *S. aureus* in an agar disk diffusion assay. Separation of the active compound was performed by a series of silica gel and Sephadex LH-20 column chromatography steps, and then the compound was further purified by preparative and semi-preparative reversed-phase HPLC. The purified active compound (15 mg) was thus obtained from *S. glabrescens.*

### 2.2. Structure Determination of Isolated Compound

The chemical structure of the active compound was studied on the basis of MS, ^1^H-, and ^13^C-NMR spectroscopic data, including HMQC, HMBC, and ^1^H-^1^H COSY experiments. The ^1^H-NMR spectrum of the active compound contained a triplet methyl protons signal at δ_H_ 0.89 and proton signals of a linear carbon chain at δ_H_ 1.29 (16H, br), δ_H_ 1.62 (2H, m) and δ_H_ 2.33 (2H, t) in a pattern typical of a laurate unit. Two methyl proton signals and one proton signal appeared at δ_H_ 1.19 (6H, dd, *J* = 7.0) and δ_H_ 2.63 (1H, m) due to an isobutyryloxy moiety, as well as two methyl proton signals and three proton signals at δ_H_ 0.93 (3H, d, *J* = 6.5), δ_H_ 1.01 (3H, d, *J* = 6.5), δ_H_ 2.16 (1H, m), δ_H_ 5.11 (1H, dd, *J* = 7.5, 7.0), and δ_H_ 5.20 (1H, d, *J* = 4.5) due to a typical methylpentanoic acid, respectively. The ^13^C-NMR spectrum revealed 22 carbons, including three ester carbonyl (δ_C_ 169.8, 173.5 and 176.3), two oxygenated methines (δ_C_ 72.0 and 76.5), five methyls [δ_C_ 13.3, 17.3, 2 (18.0) and 18.4], ten methylenes [δ_C_ 22.6, 24.9, 29.0, 29.2, 29.3, 29.4, 2 (29.6), 31.9 and 33.9] and two methines (δ_C_ 29.0 and 33.9). These facts were consistent with a molecular formula of C_22_H_40_O_6_, which was supported by HR-EIMS data (*m/z* 400.2820, [M]^+^) and ESIMS (*m/z* 423.8, [M+Na]^+^). The ^1^H-^1^H COSY spectrum of active compound showed proton correlations of δ_H_ 5.20 (H-2) with δ_H_ 5.11 (H-3); δ_H_ 5.11 (H-3) with δ_H_ 5.20 (H-2) and δ_H_ 2.16 (H-4); δ_H_ 2.16 (H-4) with δ_H_ 5.11 (H-3), δ_H_ 0.93 (H-5) and δ_H_ 1.01 (H-6); δ_H_ 0.93 (H-5) with δ_H_ 2.16 (H-4); δ_H_ 1.01 (H-6) with δ_H_ 2.16 (H-4); δ_H_ 2.63 (H-8) with δ_H_ 1.19 (H-9 and H-10). The structure of the active compound was further established according to its HMBC spectrum, in which ^1^H-^13^C long-range correlation signals were between δ_H_ 5.20 (H-2) and δ_C_ 169.8 (C-1), δ_C_ 76.5 (C-3), δ_C_176.3 (C-7); δ_H_ 5.11 (H-3) and δ_C_ 173.5 (C-1'), δ_C_ 169.8 (C-1), δ_C_ 72.0 (C-2), δ_C_ 29.0 (C-4), δ_C_ 17.3 (C-5), δ_C_ 18.4 (C-6); δ_H_ 2.16 (H-4) and δ_C_ 72.0 (C-2), δ_C_ 76.5 (C-3), δ_C_ 17.3 (C-5), δ_C_ 18.4 (C-6); δ_H_ 0.93 (H-5) and δ_C_ 76.5 (C-3), δ_C_ 29.0 (C-4), δ_C_ 18.4 (C-6); δ_H_ 1.01 (H-6) and δ_C_ 76.5 (C-3), δ_C_ 29.0 (C-4), δ_C_ 17.3 (C-5); δ_H_ 2.63 (H-8) and δ_C_ 176.3 (C-7), δ_C_ 18.0 (C-9 and C-10) (Table 1).

**Table 1:** ^1^H- and ^13^C-NMR spectral data of 3-(dodecanoyloxy)-2-(isobutyryloxy)-4-methylpentanoic acid from *S. glabrescens*.

Position	^13^C (δ)	DEPT	^1^H (δ) ^a^ (multiplicity, *J*)	^1^H-^1^H COSY	HMBC ^b^ (^1^H→^13^C)
1'	173.5	CO			
2'	33.9	CH_2_	2.33 (t, 7.0, 7.5)	H-3'	C-1', C-3', C-4'
3'	24.9	CH_2_	1.62 (m)	H-2', H-4'	C-1', C-2', C-4'
4'	29.0	CH_2_	1.29 (br)		
5'	29.2	CH_2_	1.29 (br)		
6'	29.3	CH_2_	1.29 (br)		
7'	29.4	CH_2_	1.29 (br)		
8', 9'	29.6	2(CH_2_)	1.29 (br)		
10'	31.9	CH_2_	1.29 (br)		
11'	22.6	CH_2_	1.29 (br)		
12'	13.3	CH_3_	0.90 (t, 7.0)	H-11'	C-11', C-10'
1	169.8	CO			
2	72.0	CH	5.20 (d, 4.5)	H-3	C-1, C-7, C-3
3	76.5	CH	5.11 (dd, 7.5, 7.0)	H-2, H-4	C-1', C-1, C-2, C-4, C-5, C-6
4	29.0	CH	2.16 (m)	H-3, H-5, H-6	C-3, C-2, C-5, C-6
5	17.3	CH_3_	0.93 (d, 6.5)	H-4	C-3, C-4, C-6
6	18.4	CH_3_	1.01 (d, 6.5)	H-4	C-3, C-4, C-5
7	176.3	CO			
8	33.9	CH	2.63 (m)	H-9, H-10	C-7, C-9, C-10
9, 10	18.0	2(CH_3_)	1.19 (dd, 7.0)	H-10, H-8	C-7, C-8

^a^: ^1^H directly attached to ^13^C determined from HMQC experiment; ^b^: ^1^H-^13^C long-range correlation (HMBC) corresponding to two- or three-bond connectivities.

**Figure 1:**
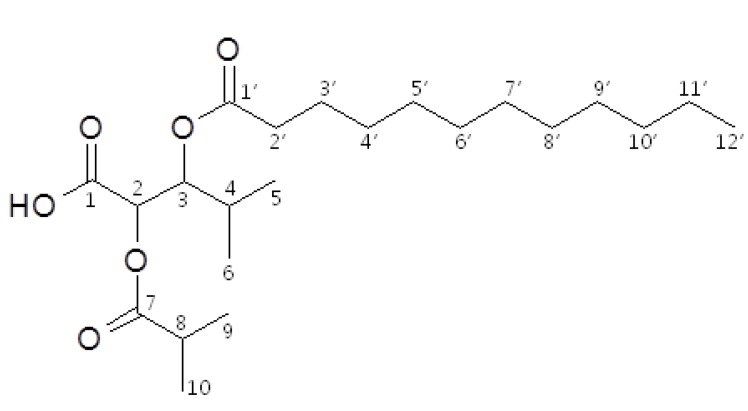
Structure of 3-(dodecanoyloxy)-2-(isobutyryloxy)-4-methylpentanoic acid from *S. glabrescens*.

From all the above information, the structure of the active compound was determined to be 3-(dodecanoyloxy)-2-(isobutyryloxy)-4-methylpentanoic acid (Figure 1), isolated from nature for the first time.

### 2.3. Antimicrobial Activity of Isolated Compound

Long-chain fatty acids are known as surface-active anionic detergents [10]. In general, fatty acid sensitivity is considered to be a characteristic of Gram-positive bacteria, with few Gram-negative species being susceptible [11]. In addition, lauric acid, which is the most effective among the saturated fatty acids, has been reported to show the antimicrobial activity against six strains of *S. aureus* [12]. In regards to the chemical structure, 3-(dodecanoyloxy)-2-(isobutyryloxy)-4-methylpentanoic acid has both the structural properties described above, as it contains a saturated long laurate chain. These structural properties suggest that the compound should display antibacterial activity against Gram-positive bacteria.

**Figure 2:**
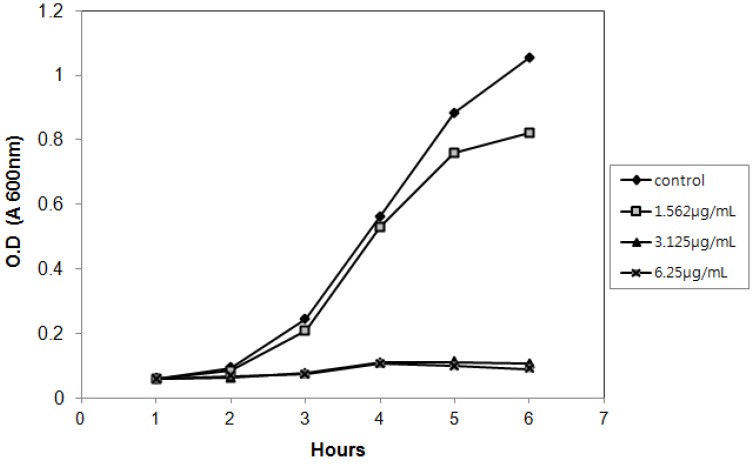
Growth rate analysis of *S. aureus* under the treatment of 3-(dodecanoyloxy)-2-(isobutyryloxy)-4-methylpentanoic acid.

The disk diffusion method was used to investigate the correlation between structure and antibacterial activity of 3-(dodecanoyloxy)-2-(isobutyryloxy)-4-methylpentanoic acid and lauric acid. As is shown in Table 2, while 3-(dodecanoyloxy)-2-(isobutyryloxy)-4-methylpentanoic acid produced an inhibition zone diameter of 11 mm against *S. aureus* at 6.25 μg/mL, no antibacterial activity was observed at the same concentration of lauric acid. An amount of 50 μg/mL of lauric acid showed a similar antibacterial activity as 6.25 μg/mL of 3-(dodecanoyloxy)-2-(isobutyryloxy)-4-methylpentanoic acid (Table 2), indicating that the efficacy of 3-(dodecanoyloxy)-2-(isobutyryloxy)-4-methylpentanoic acid against *S. aureus* was approximately 8-fold stronger than that of lauric acid. These results suggest that laurate and 2-(isobutyryloxy)-4-methylpentanoic acid may operate as key factors in the antibacterial activities against *S. aureus* and synergistically enhance the antibacterial activity. We also observed the influence of 3-(dodecanoyloxy)-2-(isobutyryloxy)-4-methylpentanoic acid on the growth of *S. aureus* at 1.562 μg/mL (1/2 MIC), 3.125 μg/mL (MIC), and 6.25 (2 MIC) μg/mL. As shown in Figure 2, 3-(dodecanoyloxy)-2-(isobutyryloxy)-4-methylpentanoic acid inhibited the growth of *S. aureus* at 3.25 μg/mL.

**Table 2:** Antibacterial activity of 3-(dodecanoyloxy)-2-(isobutyryloxy)-4-methylpentanoic acid and lauric acid against *S. aureus* measured using the disk diffusion method.

Compounds	Diameter of clear zone (mm)			
	6.25	12.50	25.00	50.00 ^a^
Lauric acid	- ^b^	-	-	11 ^c^
3-(Dodecanoyloxy)-2(isobutyryloxy)-4-methylpentanoic acid	11	12	15	19
Erythromycin	40	40	42	46

^a^: Amount of compounds (μg/mL); ^b^: No antibacterial activity; ^c^: Diameter of clear zone (mm), and diameter of filter disk is 10 mm.

Also, we further examined the antibacterial activities of 3-(dodecanoyloxy)-2-(isobutyryloxy)-4-methylpentanoic acid against Gram-positive bacteria (*S. aureus*, *B. subtilis*, *E. faecalis*, *P. acnes*, *S. epidermidis*, *S. schleiferi* subsp *. coagulans*, *S. agalactiae* and *S. pyrogens*), Gram-negative bacteria (*E. coli* and *P. aeruginosa*) and yeast strains (*C. alibicans* and *F. neoformans*) by measuring the MIC, which is the lowest concentration yielding no growth. As shown in Table 3, the MIC values of the compound for the Gram-positive bacteria were between 3.12 and 25.00 μg/mL. Specifically, while the compound showed the strongest activity against *S. aureus*, the antibacterial activity against *E. faecalis*, *P. acnes* and *S. epidermidis* was relatively low. In addition, 3-(dodecanoyloxy)-2-(isobutyryloxy)-4-methylpentanoic acid did not show any antibacterial activity against Gram-negative bacteria and yeast (Table 3). These results suggest that the antibacterial activity of 3-(dodecanoyloxy)-2-(isobutyryloxy)-4-methylpentanoic acid is specific against Gram-positive bacteria.

**Table 3:** MIC of 3-(dodecanoyloxy)-2-(isobutyryloxy)-4-methylpentanoic acid.

	Organism	MIC (μg/mL)	Organism	MIC (μg/mL)
**Gram-positive bacteria**	*B. subtilis*	6.25	*S. epidermidis*	25.00
	*E. faecalis*	25.00	*S. schleiferi*	12.50
	*P. acnes*	25.00	*S. agalactiae*	6.25
	*S. aureus*	3.12	*S. pyrogens*	6.25
**Gram-negative bacteria**	*E. coli*	- ^a^	*P. aeruginosa*	-
**yeast**	*C. alibicans*	-	*F. neoformans*	-

^a^: No activity.

## 3. Experimental

### 3.1. General

The HPLC system used was comprised of Waters prep LC 2000 a multi-solvent delivery, and Waters 2487 Dual λ Absorbance detector. UV spectra were obtained on a Dual λ Absorbance detector (Waters 2487) instrument (all Waters, Milford, MA, USA). The HPLC-grade organic solvents and bulk organic solvents were purchased from the Duksan (Ansan, Korea) and J.T. Baker (Phillipsburg, NJ, USA) companies. ^1^H- and ^13^C-NMR spectra were obtained on a Bruker Avance-500 spectrometer (Bruker Spectrospin, Rheinstetten, Germany; 500 MHz for ^1^H- and 125 MHz for ^13^C-) using methanol-*d*_4_ as solvent and tetramethylsilane (TMS) as an internal standard, and the chemical shifts were reported in δ (ppm) units relative to the TMS signal and coupling constants (*J*) in Hz. A complete attribution was performed on the basis of the 2D-experiment (heteronuclear multiple bond correlation, HMBC). Mass spectrometry (ESI) data were measured on Agilent 1100LC/MSD trap. HRMS data were recorded on a JMS-600W spectrometer (JEOL, Tokyo, Japan).

### 3.2. Plant Material

The aerial parts of *S. glabrescens* Makino (Compositae) were purchased from Sam-Hong Pharmaceutical Co., Ltd. in Seoul, Korea.

### 3.3. Extraction and Isolation

The air dried *S. glabrescens* (100 g) were cut into small pieces and extracted three times with 80% MeOH (2 L) at room temperature for 7 days, and filtered. The original 80% MeOH in H_2_O (500 mL × 3, 17.67 g) extract was evaporated to dryness *in vacuo*, and then suspended in 500 mL of water. The water suspension was partitioned three times with CHCl_3_ (100 mL). The CHCl_3_ (5.93 g) extract was evaporated to dryness *in vacuo*, and was then suspended in 500 mL of 80% MeOH in H_2_O. The 80% MeOH suspension was partitioned three times with *n*-hexane (500 mL). The 80% MeOH (4.46 g) extract was evaporated to dryness *in vacuo*, and was then suspended in 500 mL of water. The water suspension was partitioned three times with EtOAc (500 mL). The EtOAc extract (3.4 g) was evaporated to dryness *in vacuo*. Since high antibacterial activity was observed for the EtOAc extract, this extract was further investigated in detail. The EtOAc extract was chromatographed on a silica gel column (1:100 ratio of sample:silica gel), under medium pressure and eluted using a CHCl_3_-MeOH step gradient system with increasing polarity from 0%, to 2%, 4%, 6%, 8%, 10%, and 100% MeOH to give seven fractions (Fractions 1–7). Fraction 2 (1.38 g) was then subjected to Sephadex LH-20 column chromatography eluted with 80% MeOH in CHCl_3_ (5 mL/15 min) to give 80 fractions (Fractions No.1–80 at once). The Sephadex fractions 66–77 (30 mg) was further purified by preparative reversed-phase HPLC using a gradient from 75%–100% ACN in H_2_O (Luna, 250 × 21.20 mm; 5 μm particle size; 15 mL/min; UV detection at 210 nm), to afford 3-(dodecanoyloxy)-2-(isobutyryloxy)-4-methylpentanoic acid (15 mg, R_t_ 60.16 min). Oil; UV λ_max_ (MeOH) 210 nm; ^1^H-NMR and ^13^C-NMR (CD_3_OD); see Table 1; HR-EIMS (EI^+^) *m/z* 400.2820, [M]^+^ (calcd. for C_22_H_40_O_6_, 400.2829); ESIMS (*m/z*): 423.8, [M+Na]^+^ (calcd. for C_22_H_40_O_6_Na, 423.3).

### 3.4. Antibacterial Activity Assay

Gram-positive bacteria [*Staphylococcus aureus* (ATCC 6538P), *Bacillus subtilis* (ATCC 15245), *Enterococcus faecalis* (ATCC 11700), *Propionibacterium acnes* (ATCC 6919), *Staphylococcus epidermidis* (ATCC 12228), *Staphylococcus schleiferi* subsp. *coagulans* (ATCC 49545), *Streptococcus agalactiae* (ATCC 14364) and *Streptococcus pyrogens* (ATCC 19615)], Gram-negative bacteria [*Escherichia coli* (ATCC 8739) and *Pseudomonas aeruginosa* (ATCC 27853)] and yeast [*Candida alibicans* (ATCC 10231) and *Filobasidiella neoformans* (ATCC 34144)] were used in these experiments. The antibacterial activity was determined using both the agar diffusion and broth dilution techniques as described previously by Cheesbrough [13] and Gatsing *et al*. [14]. Agar diffusion susceptibility testing was performed using the disc method. A disc of blotting paper was impregnated with 50 μL of a 60 mg/mL (for crude extract) or 4 mg/mL (for pure compounds) solution of each sample dissolved in DMSO. Thus, the disc potencies were 1 mg and 200 μg for the crude extract and pure compounds, respectively. Erythromycin (Sigma, St. Louis, MO, USA) was used as the standard drug. After drying, the disc was placed on a plate of sensitivity testing agar inoculated with the test organism. Petri dishes were left at room temperature for approximately 45 min to allow the extract or the compounds to diffuse from the disc into the medium, and were then incubated at 37 °C for 24–48 h. The zones showing no growth were then noted and their diameters were recorded as the zones of inhibition.

We pre-cultured the bacterial cells for 24 h at 37 °C in 10 mL broth. Approximately 5 × 10^5^ cfu/mL bacterial cells of the pre-cultured bacteria were inoculated into 3 mL of broth. The samples were then added into approximately 3 mL of broth containing the bacteria and cultured for 24 h at 37 °C. To determine the activity of the samples, we employed a two-fold serial dilution method. The total volume of the mixture was approximately 3 mL, with the test-compound concentrations in the tube ranging from 200 to 0.78 μg/mL and the concentration of standard compound (erythromycin) ranged from 100 to 0.78 μg/mL. After 24 h of incubation at 37 °C, the MIC value was defined as the lowest concentration that inhibited the visible growth of tested microorganism.

## 4. Conclusions

It was demonstrated that a new compound, 3-(dodecanoyloxy)-2-(isobutyryloxy)-4-methylpentanoic acid isolated from *S. glabrescens* has antibacterial activities specifically against Gram-positive bacteria. We found that the antibacterial effect of 3-(dodecanoyloxy)-2-(isobutyryloxy)-4-methylpentanoic acid was due to both the 2-(isobutyryloxy)-4-methylpentanoic acid group and laurate group. In addition, the antibacterial activity of 3-(dodecanoyloxy)-2-(isobutyryloxy)-4-methylpentanoic acid was more potent than lauric acid itself. To the best of our knowledge, this is the first study demonstrating the antibacterial activity of *S. glabrescens*. In addition, we demonstrated that 3-(dodecanoyloxy)-2-(isobutyryloxy)-4-methylpentanoic acid is an antibacterial compound of *S. glabrescens*. The compound also has a specific antibacterial activity against Gram-positive bacteria and the strongest activity was observed against *S.aureus*, which are major foodborne pathogenic microorganisms.
